# Work characteristics and health in older workers: Educational inequalities

**DOI:** 10.1371/journal.pone.0241051

**Published:** 2020-10-23

**Authors:** Sascha de Breij, Martijn Huisman, Dorly J. H. Deeg

**Affiliations:** 1 Department of Epidemiology and Data Science, Amsterdam Public Health Research Institute, Amsterdam UMC, Vrije Universiteit Amsterdam, Amsterdam, The Netherlands; 2 Department of Sociology, Vrije Universiteit Amsterdam, Amsterdam, The Netherlands; University of Botswana, BOTSWANA

## Abstract

To be able to extend working lives, maintaining good health in older workers is important. The aim of the present study was to identify which work characteristics are associated with physical and mental health outcomes in older workers in the Netherlands, and particularly whether there are educational differences in these associations. We used longitudinal tobit and ordered logistic regression analyses to examine the associations between physical demands, psychosocial demands, variation in tasks, autonomy, and job strain and self-rated health (SRH), functional limitations, and depressive symptoms. We included interaction terms between the work characteristics and education to examine effect modification by education. We found that high physical demands, low variation in tasks, low autonomy, and high job strain were associated with poorer physical and mental health. We found evidence for educational differences in the exposure to these work characteristics, as well as in the strengths of their associations with health, with lower educated workers being disadvantaged. The associations between physical demands (SRH: OR = 3.70 (95%CI:1.92;7.11); functional limitations: B = 1.27 (95%CI:.47;2.07)), autonomy (SRH: OR = .42(95%CI:.26;.69)), and job strain (active job; SRH: OR = .25 (95%CI:.09;.69); functional limitations: B = -1.51 (95%CI:-2.68;-.34), and health were strongest in the lower educated workers. In order to maintain good health in older workers and reduce health inequalities, it is recommended to implement workplace interventions to improve working conditions, especially among the lower educated workers.

## Introduction

Because of longer life expectancy and low birth rates, the proportion of older workers (50–64 years) in the workforce will be double the size of younger (<25 years) workers by the year 2025 in de EU15 countries [1]. Maintaining good health in this particular group of workers is therefore becoming increasingly important. Many studies have shown that poor physical and mental health are associated with a loss of productivity at work [2], reduced workability [e.g. 3], more sickness absence [e.g. 4], and early exit from paid work [e.g. 5]. In the Netherlands, work-related costs, i.e. costs due to absenteeism, disability pensions, and health care, are estimated to be around €8,7 billion each year [6]. With more older workers who are having to spend more years in the workforce due to an increasing statutory retirement age, these numbers may increase in the coming decades. To implement workplace interventions to improve health, it is necessary to identify which modifiable factors are associated with poor health.

**Fig 1:**
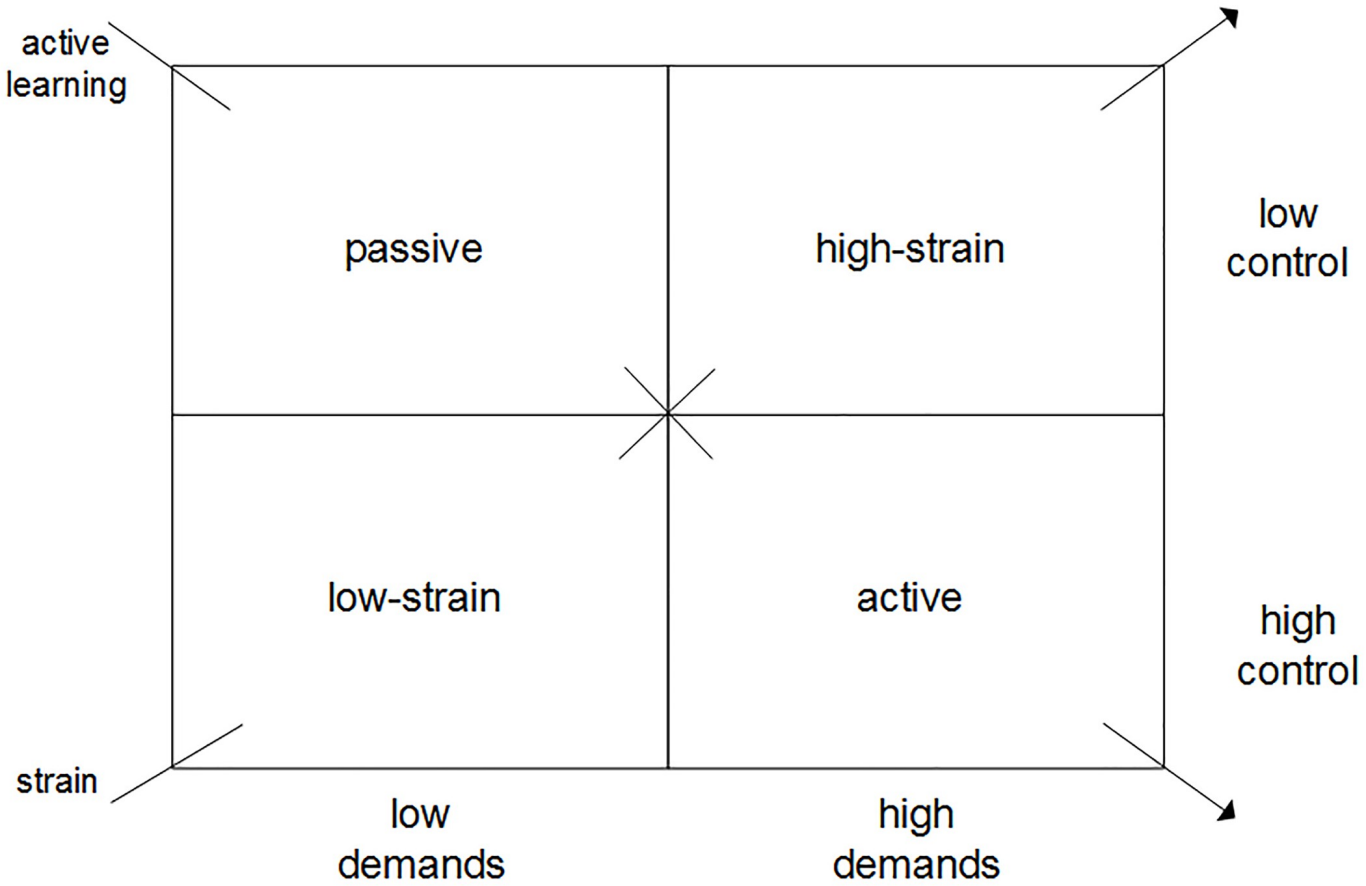
The four job types following Karasek’s job demand control model.

Physical and psychosocial work demands as well as resources at work, such as autonomy at work, variation in tasks, and social support, have been shown to be associated with both physical and mental health outcomes [7–10]. One of the most influential models in this context is Karasek’s Job Demand-Control (JDC) model [11,12]. In this model, not only the individual work characteristics are considered in relation to well-being, but also their interrelation. A composite measure derived from psychosocial job demands and job control is called ‘job strain’. Four types of jobs can be distinguished based on job strain, as shown in Fig 1: high-strain jobs (high demands and low control), low-strain jobs (low demands and high control), active jobs (high demands and high control) and passive jobs (low demands and low control). Demands are not necessarily negative; under the right circumstances they can also have a positive effect on well-being. It is hypothesized that in active jobs, job demands actually lead to active learning and motivated behavior, because these workers have the required job control, turning the demands into a positive job aspect. Passive jobs on the other hand may lead to a lack of motivation and reduced acquisition of knowledge.

Low educated people generally have more health problems than the higher educated [13,14] and there is some evidence that these inequalities have increased over the last decades [15–17]. A low education is associated with more adverse working conditions [18,19], with the exception of psychosocial job demands, which are more common among workers with a high educational level [19–21]. Furthermore, low educated people need to work more years until they are entitled to state pensions, compared to their higher educated counterparts, who spend more years in school before entering the labour market [22]. Thus, much is known about the differences in work characteristics across educational groups.

But not only are there educational differences in the exposure to adverse working conditions, there may also be educational differences in the effects of these work characteristics on health. Workers with a high educational level generally have more material and psychosocial resources, e.g. a greater sense of mastery, optimism and social support [23,24], that may help them to better cope with adverse working conditions. Thus, adverse working conditions may have less impact on the health of higher educated workers compared to their lower educated peers. However, research on this moderating effect of education is scarce. The few studies that did examine educational differences, have shown evidence that associations between job strain and self-rated health, angina, and myocardial infarction are indeed less strong in higher educated compared to lower educated workers [25].

To increase workability and decrease the risk of early work exit in older workers, identification of modifiable work characteristics is necessary. Examining whether the health effects of these work characteristics differ across educational groups facilitates the development and implementation of targeted interventions to maintain good health in all older workers. While a lot of research has been done on the associations between work characteristics and health, most studies have focused on the general working population. Research on the growing group of older workers is scarce. In addition, educational inequalities in these associations are often neglected. The aim of the present study is to identify which work characteristics are associated with physical and mental health outcomes in older workers. We examine potential effect modification by education of these associations. Our research questions are:

1. Which work characteristics are associated with physical and mental health in older workers?
2. Are there educational differences in these associations, i.e. is education an effect modifier?

## Methods

### Sample

We used data from the Longitudinal Aging Study Amsterdam (LASA). LASA is an ongoing, prospective cohort study in the Netherlands on the determinants, trajectories and consequences of physical, cognitive, emotional, and social functioning in older adults. Measurements are conducted approximately every three years. Sampling, response and procedures are described in detail elsewhere [26]. LASA received approval by the medical ethics committee of the VU University Medical Center. All data were fully anonymized before accessing them. Respondents provided informed written consent to have their data used in research. Data from the first (respondents aged 55–84 entering the study in 1992–1993), second (new respondents aged 55–64 entering the study in 2002–2003), and third (new respondents aged 55–64 entering the study in 2012–2013) cohorts were pooled for the current study and all waves through 2016 were included. We included every participant who had a paid job at baseline and was younger than the statutory retirement age (n = 1295). Follow-up measurements were included until respondents reached the statutory retirement age, exited the workforce, or dropped out of the study, whichever occurred earlier. All time-varying variables were measured longitudinally, with the number of follow-up measurements depending on the time of entering the study and loss to follow-up.

### Measures

#### Outcomes

We included three health indicators as outcomes, measuring different aspects of physical and mental health. Self-rated health (SRH) can be seen as a global measure of people’s perception of their health [27]. Functional limitations are restrictions in the ability to perform activities of daily living (ADL). Depressive disorders are among the most common mental health conditions and their global prevalence has increased in recent decades [28].

#### Self-rated health

SRH was measured with the question ‘How do you rate your health in general?’ with response options (1) very good, (2) good, (3) fair, (4) sometimes good/sometimes poor, and (5) poor.

#### Functional limitations

This measure consists of six items: cutting one’s own toenails, dressing and undressing oneself, sitting down and standing up from a chair, walking outside for five minutes without stopping, walking up and down a staircase of 15 steps without resting, and use of own or public transportation. Five response options range from ‘yes, without difficulty’ to ‘no, I cannot’. A sum score is calculated by counting the number of items ’with some difficulty’ or worse (range 0–6). A higher score reflects more functional limitations.

#### Depressive symptoms

Depressive symptoms were measured with the Center for Epidemiologic Studies Depression Scale (CES-D) [29]. The scale consists of 20 items covering depressive symptomatology experienced in the past week. There are four response options ranging from 0 ’rarely or never’ to 3 ’mostly or always’. The sum score ranges from 0 to 60, with higher scores indicating more depressive symptoms.

#### Independent variables

Work characteristics were derived from a general population job exposure matrix (GPJEM) for 55 to 65 year olds [30]. The GPJEM indicates levels of exposure probability of physical and psychosocial demands and psychosocial resources, based on occupational category. Because of non-linear associations with our outcomes, we dichotomized all continuous work characteristics into low and high exposure, based on the median.

#### Physical demands

For physical demands, a sum score of three items was assigned to each occupational category: use of force, uncomfortable work, and exposure to repetitive movements.

#### Psychosocial demands

For psychosocial work demands, a sum score of three items was used: time pressure (work at high pace and work under high time pressure), task requirements (work fast, much work, work hard, and hectic work) and cognitive demands (intensive thinking, need to keep focused, and requiring much concentration).

#### Autonomy

Autonomy at work was measured with the following items: decide how to perform the job, the sequence of tasks, work pace, and when to take time off.

#### Variation in tasks

Variation in tasks consisted of three items: variation in work, learn new things, and work requires creativity.

#### Job strain

Job strain was categorized according to the quadrant approach [11], using the aforementioned measures of psychosocial demands and autonomy (control): low-strain (low demands/high control); active (high demands/high control); passive (low demands/low control); and high-strain (high demands/low control).

#### Control variables

We controlled for sex, age, year, region, and the number of working hours. Because the association between number of working hours and health was not linear, we categorized it into four categories representing the most common part-time, full-time and more than full-time working hours: 1–15; 16–31; 32–40; ≥41. To answer our research question, effect modification by educational level was investigated. For educational level, the International Standard Classification of Education 2011 (ISCED 2011) was used. We categorized educational level into three groups: low (up to lower secondary education, ISCED 0–2), intermediate (upper secondary education or post-secondary non-tertiary education, ISCED 3–4) and high (short cycle tertiary and higher, ISCED 5–6).

### Statistical analysis

To examine the associations between the work characteristics and SRH, we conducted random-effects ordered logistic regression analyses, to take into account the clustering in the data due to repeated measures and the ordinal nature of the outcome variable. Because a large number of respondents had a score of zero on the functional limitations and depressive symptoms scales, random-effects tobit regression models were fit. Tobit regression takes into account this left-censoring of the data at the lower level of the outcome variables [31].

We built regression models for each independent variable and each outcome. We stratified by educational level and tested interactions between the work characteristics and education to examine whether educational differences were statistically significant (p < .10 [32]). If there was no statistical support for educational differences, education was included as a confounder and effects in the total sample were evaluated.

## Results

Table 1 shows the characteristics of our sample. Workers with a low and intermediate educational level had more depressive symptoms, more functional limitations, and poorer SRH than higher educated workers. In the lower educated group, the percentage of workers with high physical demands was much higher compared to the higher educated group, whereas psychosocial demands, variation in tasks, and autonomy were more common in the higher educated workers.

**Table 1:** Characteristics of the sample.

	Low education (n = 575)	Intermediate education (n = 306)	High education (n = 414)	Total (n = 1295)
Men (%)	55.0	56.9	64.3	58.5
Age at baseline (M (SD))	58.4 (2.4)	58.9 (2.7)	58.5 (2.5)	58.5 (2.5)
Physical demands, above median (%)	84.9	70.1	28.0	62.7
Psychosocial demands, above median (%)	20.6	36.3	83.0	44.9
Variation in tasks, above median (%)	8.8	26.4	73.6	34.3
Autonomy, above median (%)	35.9	45.1	65.4	44.8
Job strain (%)				
Low-strain	28.4	29.9	14.2	24.0
Passive	51.6	34.7	6.4	32.7
Active	9.7	16.6	44.1	22.7
High-strain	10.3	18.8	35.3	20.6
SRH (%)				
Very good	15.8	17.4	26.8	19.9
Good	59.2	59.5	59.7	59.5
Fair	16.5	15.8	10.4	14.3
Sometimes good/sometimes poor	7.9	6.6	1.9	5.6
Poor	0.7	0.6	1.1	0.8
Depressive symptoms (M (SD))	5.9 (5.8)	6.1 (6.1)	5.3 (5.4)	5.7 (5.8)
Functional limitations (M (SD))	0.5 (0.9)	0.5 (0.9)	0.3 (0.7)	0.4 (0.9)

Notes: M = mean, SD = standard deviation.

In workers with a low and intermediate education, jobs characterized by low-strain and passive jobs were most common. In the higher educated, active jobs and high-strain jobs were most common.

In Table 2 the associations between the work characteristics and the health outcomes can be found. High physical demands were associated with poor health and effect modification by education was evident. The associations between physical demands and SRH and functional limitations were stronger in the low educated workers compared to workers with an intermediate and high education. The OR of 3.70 for SRH indicates that low educated workers with high physical demands have 3.7 times the odds of poor SRH compared to low educated workers with low physical demands. The B of 1.27 for functional limitations indicates that low educated workers with high physical demands on average have 1.27 more functional limitations than low educated workers with low physical demands. The association of physical demands and depression was statistically significant only in the intermediate education level, however, the interaction term of education and physical demands was not statistically significant.

**Table 2:** Associations between work characteristics and SRH, functional limitations, and depression.

	SRH OR (95% CI)^a^				Functional limitations B (95% CI)^a^				Depressive symptoms B (95% CI)^a^			
	Low education	Intermediate education	High education	Total	Low education	Intermediate education	High education	Total	Low education	Intermediate education	High education	Total
Physical demands (below median = ref. cat.)	3.70 (1.92;7.11)**	**.90 (.43;1.91)**	**1.47 (.74;2.93)**	1.85 (1.24;2.74)**	1.27 (.47;2.07)**	**.12 (-.64;.88)**	**-.03 (-.07;.65)**	.40 (-.02;.82)†	.62 (-.71;1.94)	1.55 (.07;3.03)*	-.36 (-1.55;.82)	.56 (-.20;1.32)
Psychosocial demands (below median = ref. cat.)	.35 (.19;.66)**	.63 (.32;1.24)	.49 (.20;1.21)	.48 (.32;.72)**	-.59 (-1.24;.06)	**.17 (-.55;.88)**	-.38 (-1.17;.41)	-.22 (-.63;.19)	.35 (-.88;1.58)	-.43 (-1.83;.97)	.13 (-1.27;1.53)	.01 (-.76;.78)
Variation in tasks (below median = ref. cat.)	.43 (.18;1.05)	.73 (.34;1.59)	.58 (.27;1.24)	.60 (.38;.94)*	.06 (-.85;.97)	-.02 (-.82;.79)	-.25 (-.91;.42)	-.05 (-.51;.41)	.59 (-1.13;2.30)	-.44 (-2.00;1.11)	.54 (-.66;1.75)	.26 (-.58;1.11)
Autonomy (below median = ref. cat.)	.42 (.26;.69)**	.85 (.42;1.71)	**.81 (.43;1.53)**	.61 (.43;.85)**	-.73 (-1.29;-.17)*	-.21 (-1.01;.58)	-.11 (-.72;.49)	-.39 (-.75;-.02)*	-.55 (-1.60;.50)	-.84 (-2.29;.60)	-.73 (-1.83;.37)	-.68 (-1.35;-.00)*
Job strain (high strain = ref. cat.)												
Active	.25 (.09;.69)**	**1.70 (.56;5.19)**	**.78 (.39;1.53)**	.64 (.39;1.03)†	-1.51 (-2.68;-.34)*	**.32 (-.86;1.49)**	**-.32 (-.97;.34)**	-.46 (-.97;.06)†	-1.03 (-3.18;1.12)	-.92 (-3.19;1.34)	-1.18 (-2.35;-.02)*	-1.18 (-2.12;-.24)*
Low-strain	.98 (.41;2.35)	1.53 (.60;3.91)	1.37 (.54;3.47)	1.24 (.76;2.01)	-.47 (-1.34;.40)	-.36 (-1.47;.74)	.25 (-.64;1.15)	-.27 (-.80;.27)	-1.20 (-2.95;.56)	-.22 (-2.22;1.77)	-.78 (-2.37;.80)	-.81 (-1.79;.16)
Passive	1.75 (.76;4.07)	2.57 (1.01;6.55)*	2.78 (.69;11.26)	2.13 (1.29;3.51)**	.14 (-.69;.96)	.18 (-.75;1.12)	-.24 (-1.47;.98)	.15 (-.36;.66)	-.70 (-2.40;1.00)	.32 (-1.55;2.19)	-1.55 (-3.62;.52)	-.55 (-1.52;.41)

^†^ p < .10;

* p < .05;

** p < .01.

^a^ OR/B adjusted for age, year, number of working hours, region, and sex. OR’s/B’s in bold differ statistically significantly (p < .10) from the OR’s/B’s in the low education group (= ref.cat).

High psychosocial demands were associated with better SRH. No statistically significant association of psychosocial demands and functional limitations or depression was observed.

Among the psychosocial resources, high variation in tasks was associated with better SRH, regardless of educational level, but not with functional limitations and depression (Table 2). High autonomy was associated with better SRH, in the low educated group most strongly, and with fewer functional limitations and less depressive symptoms, regardless of educational level.

Regarding job strain, low educated workers with an active job reported better SRH and less functional limitations than their counterparts with a high-strain job. We found a similar protective effect of active jobs for depressive symptoms, regardless of education. For all workers, having a passive job was associated with poorer SRH.

## Discussion

The aim of our study was to identify which work characteristics are associated with health in older workers across educational levels. We examined three health indicators: SRH, depressive symptoms, and functional limitations. Previous studies have shown that high physical and psychosocial work demands, less psychosocial resources, and high job strain are associated with poor health [7,8,19,33–35]. Although most research focused on the general working population there is some evidence that these associations are stronger in older workers [36,37]. In line with previous research, we found that high physical demands were associated with poorer SRH and more functional limitations. However, educational differences in these associations were evident, with strongest effects in the low educated workers. We did not find evidence that the health of high educated workers was affected by physical demands. Jobs with high physical demands in low educated workers (e.g. construction workers) are rather different to jobs with high physical demands in highly educated workers (e.g. surgeons), which may explain these educational differences. Furthermore, higher educated workers may have more resources to better cope with their job demands.

Contrary to previous studies, we found that high psychosocial demands were associated with better SRH. This contradiction may be due to the use of different measures of psychosocial demands. In our study, psychosocial demands were mainly operationalized as cognitive demands, e.g. doing tasks that require a lot of concentration and working fast and under time pressure. Most studies have used Karasek’s Job Content Questionnaire [38] or similar measures to operationalize psychosocial job demands. These measures contain more negative items compared to ours, such as having conflicting demands and having insufficient time to work [38]. Our measure of psychosocial job demands may reflect more cognitive demands that are beneficial for health rather than stressors. In addition, by using a job exposure matrix, our measure of the work characteristics was objective, while most studies have used self-reported measures of work demands, which increases the risk of reversed causality, because unhealthy respondents may report higher demands [39].

When examining psychosocial resources, we found that, regardless of educational level, high variation in tasks was associated with better SRH and high autonomy was also associated with better SRH, fewer functional limitations, and fewer depressive symptoms. These findings are in line with previous research [7,19]. The positive effect of autonomy on SRH and functional limitations was strongest in the lower educated workers.

Finally, we examined the association between job strain and health. With regard to SRH, functional limitations and depression, having an active job was associated with better health than having a high-strain job. These results support the hypothesis that the combination of high demands and high control leads to active learning and motivation and is beneficial for one’s health. Generally, this effect was most prevalent in the lower educated workers.

Thus, not only are low educated workers exposed more often to high physical job demands and less psychosocial resources, and less often have active jobs compared to high educated workers, the health effects of these work characteristics are also stronger in lower educated workers. These findings are in agreement with previous studies that found socioeconomic differences in the relation between job strain and health [25]. This modifying effect of education might be explained by differences in resources. Low educated workers generally have less material and psychosocial resources that may help them to cope with adverse working conditions [23,40]. Our results suggest that these resources may play less of a role when it comes to depressive symptoms, for which we did not find a modifying effect of education.

Our study has some limitations. Working conditions can change multiple times over the career. Changes that took place before entering our study have not been taken into account. It could be that workers with poor health already changed jobs to better accommodate their health problems. This may have led to an attenuation of observed associations between work characteristics and health. Another limitation is the possibility of a healthy worker effect, i.e. workers with severe health problems may have already left the workforce prior to entering our study. Those still working may have less severe limitations, which may also attenuate our results.

Our study also has important strengths. We are among the first to focus on educational differences in the associations between work characteristics and health in older workers. Especially in the Netherlands there has been a lack of information on educational inequalities in older workers [41]. So far, educational level has usually been included as a confounder, but the modifying role of education has largely been neglected. Also, we included a sample of male and female workers with measurements up to work exit. Furthermore, we included objective measures of work characteristics, thereby reducing the risk of reversed causality. The disadvantage of using a job exposure matrix, however, is that heterogeneity within occupations is not taken into account.

Our findings highlight the importance of improving work characteristics in order to maintain good health in older workers in general, but also to reduce educational inequalities in health. With having to spend more years working due to an increase in the statutory retirement age, our results indicate that it is important to adapt working conditions. To reduce physical work demands, participatory ergonomics interventions seem to be promising [42]. Interventions that aim to increase autonomy at work are promising in reducing sick leave and increasing productivity [43].

In conclusion, we found that physical demands, variation in tasks, autonomy, and job strain were associated with physical and mental health outcomes. Educational differences were not only present in the exposure to these work characteristics, but in some occasions also in the strengths of their associations with health, with lower educated workers being disadvantaged. Evidence for this was observed for the associations between physical demands, autonomy, and job strain and the health outcomes SRH and functional limitations. In order to maintain good health in older workers and reduce health inequalities, it is recommended to implement workplace interventions to improve working conditions. Targeting physical demands, autonomy, and job strain may especially benefit the lower educated workers.
